# Determination of the Main Phase Transition Temperature of Phospholipids by Nanoplasmonic Sensing

**DOI:** 10.1038/s41598-018-33107-5

**Published:** 2018-10-04

**Authors:** Wen Chen, Filip Duša, Joanna Witos, Suvi-Katriina Ruokonen, Susanne K. Wiedmer

**Affiliations:** 1 Department of Chemistry, POB 55, 00014 University of Helsinki, Helsinki, Finland; 2 Institute of Analytical Chemistry of the Czech Academy of Sciences, Veveří 97, Brno, 60200 Czech Republic; 3 Department of Bioproducts and Biosystems, POB 16300, 00076 Aalto University, Espoo, Finland

## Abstract

Our study demonstrates that nanoplasmonic sensing (NPS) can be utilized for the determination of the phase transition temperature (T_m_) of phospholipids. During the phase transition, the lipid bilayer undergoes a conformational change. Therefore, it is presumed that the T_m_ of phospholipids can be determined by detecting conformational changes in liposomes. The studied lipids included 1,2-dipalmitoyl-*sn*-glycero-3-phosphocholine (DPPC), 1,2-dimyristoyl-*sn*-glycero-3-phosphocholine (DMPC), and 1,2-distearoyl-*sn*-glycero-3-phosphocholine (DSPC). Liposomes in gel phase are immobilized onto silicon dioxide sensors and the sensor cell temperature is increased until passing the T_m_ of the lipid. The results show that, when the system temperature approaches the T_m_, a drop of the NPS signal is observed. The breakpoints in the temperatures are 22.5 °C, 41.0 °C, and 55.5 °C for DMPC, DPPC, and DSPC, respectively. These values are very close to the theoretical T_m_ values, *i*.*e*., 24 °C, 41.4 °C, and 55 °C for DMPC, DPPC, and DSPC, respectively. Our studies prove that the NPS methodology is a simple and valuable tool for the determination of the T_m_ of phospholipids.

## Introduction

A liposome is a vesicle composed of phospholipids with a structure similar to the biological membrane. Because of this, liposomes have been widely applied in biological research as a biomimetic membrane^1,2^. Interactions of target compounds with liposomes have been used to predict the effect of compounds on cell membranes^3–6^. Permeability is an important physical property of liposomes, because it affects the movement of target compounds through the cell membranes. Another important property of liposomes is their applicability in drug delivery, and specific types of liposomes are used to encapsulate the functional ingredients of drugs^7–12^. The permeability of liposomes increases when the bilayer transforms from an ordered gel phase to a disordered fluid phase at the main phase transition temperature (T_m_). The T_m_ has an important effect on the structure of the biomembrane. When the phospholipids are below their specific T_m_, the individual phospholipids are closely packed, and they form a rigid ordered gel phase^13,14^. When the temperature gets close to the T_m_, the tightly packed phospholipids start to loosen up, resulting in more empty space between the phospholipids. The structure of the biomembrane becomes looser until a disordered fluid crystalline phase is established at the end. Additionally, the phase state of phospholipids has an influence on substrate-induced (titanium oxide and silicon oxide) shape deformation of liposomes^15^.

The traditional technique for determining lipid phase transitions is differential scanning calorimetry (DSC)^16–19^. The principle of the methodology is based on changes in the thermal properties of the phospholipids during the phase transition. Heat absorption occurs when the liposomes undergo phase transition from an ordered gel phase to a disordered fluid crystalline phase. The heat absorption is recorded as a heat flow in DSC as a function of the temperature. The temperature recorded at maximum heat flow is defined as the T_m_ of the lipid.

Although the T_m_ is linked to the permeability of liposomes^20,21^, the connection between them is indirect. This is because the determination of the T_m_ by DSC is based on the thermal properties of the liposomes, and not on the conformational change of the liposomes. The permeability of the lipid bilayer is known to be enhanced above the T_m_, but it has been demonstrated in a previous study that the diffusion rate of labelled compounds through 1,2-dipalmitoyl-*sn*-glycero-3-phosphocholine (DPPC) was higher before reaching the T_m_ than at the specific T_m_^20^. In addition, it has been shown that the temperature, at which labelled compounds were released from DPPC liposomes, was lower than the T_m_ of DPPC^22^. This suggests that a conformational change in the liposomes during phase transition happens before the temperature reach the T_m_. To get a better understanding of the connection between the T_m_ and the structural transformations of liposomes, it would be beneficial to detect the liposome conformational change at the same time when liposomes undergo phase transition. This could also be helpful in the future for the development of thermosensitive liposomes for drug delivery.

Recently, quartz crystal microbalance with dissipation monitoring (QCM-D)^23–25^ and nanoplasmonic sensing (NPS) have been widely applied in the investigation of liposome properties^26–34^. The methodologies are highly attractive as they are label-free detection techniques and have high sensitivity for biomembrane measurements. In QCM-D, the detected signal includes resonance frequency and energy dissipation. A change of frequency and dissipation is an indication for a change in the mass and viscoelasticity of a layer adsorbed on a sensor surface. QCM-D has previously been used to investigate the phase transition of adsorbed lipid vesicles^35^ and single supported phospholipid bilayers (SPBs)^36,37^. The intrinsic dependence of the crystal resonance frequency on the temperature demands sophisticated evaluation of the data (*i*.*e*., first-order derivative of dissipation over temperature profile analysis)^38^. Temperature cycling is usually used to detect hysteresis of the phase transition temperature and requires perfectly controlled temperature gradients of the measurement cell. As the phase transition is not connected with a direct change in the adsorbed biomembrane mass, the use of QCM for detecting the T_m_ relies on the analysis of vesicle/biomembrane deformation and change in the viscoelastic parameters^38–41^. Using such an approach the authors were able to define T_m_s correlating very well with those obtained by traditional T_m_ determination methods (*e*.*g*. DSC). Nanoplasmonic sensing (NPS) with lower intrinsic dependence temperature change, considered as a complimentary technique to QCM-D, is a technique of high potential for detecting small structural changes in biomaterials^15,42^. NPS is an optical technique based on localized surface plasmon resonance (LSPR) phenomenon, which is often used for nanomaterial biosensing^43–46^. The main detected signal in NPS is the maximum extinction wavelength, λ_max_. A change in the λ_max_ (peak shift ∆λ) might indicate a change of the shape of liposomes adsorbed on the sensor surface^34^. A decay length of the evanescent field of around 20 nm or less^47^ determines the method for studies of changes, happening in very close proximity of the sensing surface. We hypothesize that when adsorbed liposomes undergo phase transition, the membrane surface area deforms (expansion or contraction). This could result from the change of vesicle bending energy Q ≡ Wr^2^/κ (W: contact energy per unit area; κ: membrane bedding rigidity; r: vesicle radius in solution)^15,48,49^, since the temperature affects both κ and W. NPS has been used for temperature-dependent measurements^15,42^. A previous study successfully demonstrated that NPS has the ability to observe differences in the deformation of adsorbed vesicles at low surface coverage in their gel and fluid phases, respectively^15^. Although the behavior of close-packing of adsorbed liposomes during phase transition would be more complicated, we assume that NPS could have the potential to detect the main phase transition by analysis of the conformational change in liposomes near the sensor surface.

The aim of this study was to demonstrate the applicability of NPS to the determination of the T_m_ of phospholipids, including DPPC, 1,2-dimyristoyl-*sn*-glycero-3-phosphocholine (DMPC), and 1,2-distearoyl-*sn*-glycero-3-phosphocholine (DSPC). Additionally, to further explore the potential application of NPS in differentiating liposome properties in different phases, an ionic liquid (IL), [P_14444_][OAc], was used as a model compound. Our previous study has shown the effect of [P_14444_][OAc] on the fluid phase liposomes^50^. In this study, we further compared the interactions of [P_14444_][OAc] with both gel and fluid phase liposomes. To our knowledge, this is the first study applying NPS to determine the T_m_ of phospholipids and to detect ionic liquid interactions with liposomes in different phases.

## Results and Discussion

### Immobilization of liposomes

DPPC liposomes were prepared in three different ways: by extrusion or by sonication for 20 min or 40 min. The size distributions (diameters) by number of the extruded DPPC liposomes (97 ± 4 nm) were larger than those of the sonicated liposomes (83 ± 13 nm and 72 ± 9 nm) (Table 1).

**Table 1:** The transition temperature^18,57^, liposome size (diameter) by number, immobilization time, and peak shift for DLPC, DMPC, DPPC, and DSPC liposomes.

Phospholipids	Acyl chain length	T_m_ (°C)	Preparation method	Liposome size (nm)	Immobilization time (min)	Peak shift ∆λ (nm)
DLPC	C12	−2	20 min Sonication	32 ± 5	15	2.6
DMPC	C14	24	20 min Sonication	46 ± 15	15	2.9
DPPC	C16	41	Extrusion	97 ± 4	30	4.7
			20 min Sonication	83 ± 13	25	4.7
			40 min Sonication	72 ± 9	25	4.8
DSPC	C18	55	20 min Sonication	75 ± 7	30	4.1

**Figure 1:**
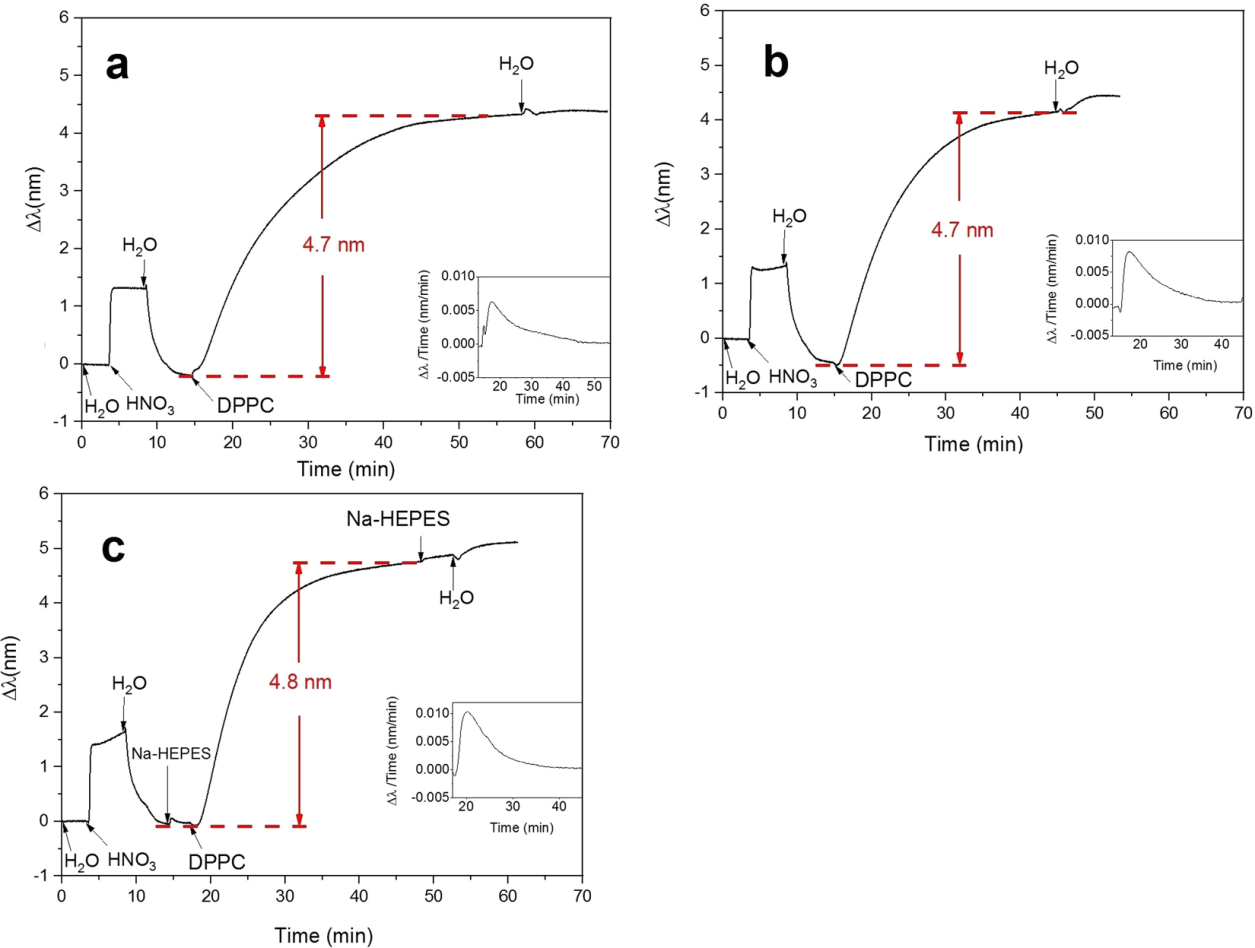
Immobilization of DPPC prepared in three different ways. (**a**) Extrusion, (**b**) sonication for 20 min, and (**c**) sonication for 40 min. The insets of figures demonstrate the peak shift time derivative curves.

The immobilization procedure of DPPC liposomes on the silicon dioxide (SiO_2_) sensor was conducted at 25 °C, at which DPPC is in the gel phase (T_m_ of 41 °C). The NPS signals (peak shift) were recorded starting from the pretreatment of the sensor surface until complete immobilization of liposomes (Fig. 1). After the HNO_3_ pretreatment, DPPC liposomes were introduced into the measurement chamber. An increase in the peak shift was observed for both extruded and sonicated DPPC liposomes, suggesting that DPPC liposomes were immobilized on the SiO_2_ sensor surface. The increase in the peak shifts for both extruded and sonicated liposomes were 4.7–4.8 nm (Fig. 1). After the immobilization, water was introduced to rinse the sensor. No drop in the peak shift was observed, meaning that the DPPC liposomes were strongly immobilized on the SiO_2_ sensor surface. A small increase in the peak shift was observed with sonicated liposomes after the water rinse, which might indicate that the sonicated liposomes were able to rearrange to cover the sensor surface more densely. Although the peak shifts were similar using either extruded or sonicated DPPC liposomes, we observed that the immobilization time for the extruded liposomes (30 min) was longer than for the sonicated liposomes (25 min). The explanation to this can be found in a recent study, showing that the larger the liposomes the longer is the time needed for their immobilization^51^.

The peak shift time derivative curve for each liposome immobilization procedure are shown in the insets of Fig. 1. The peak shift time derivative values gave indications about two aspects. The first aspect is the rate of liposome immobilization. The highest point of peak shift time derivative for sensor immobilization with extruded liposomes was 0.007 nm/min, which was lower than the value observed using sonicated liposomes (around 0.010 nm/min), correlating with shorter immobilization time using sonicated liposomes. The second aspect is related to information about the type of immobilized layers: a supported lipid bilayer (SLB) or a supported vesicle layer (SVL). The formation of a SLB is usually indicated by a sudden acceleration of the peak shift change during the liposome immobilization procedure^52^. The pattern of the derivative curve obtained in Fig. 1 did not show any acceleration of the immobilization and was confirmed as a SVL formation pattern^51^. The peak shifts of immobilized extruded or sonicated DPPC liposomes were similar (as discussed above), but since the liposome immobilization rate of sonicated liposomes (0.010 nm/min) was higher that of extruded liposomes (0.007 nm/min), we used sonication to prepare the liposomes for the following studies.

DPPC liposomes were dispersed in buffer solution (HEPES; Fig. 1c), hence the sensor surface was rinsed with HEPES before introducing the liposomes. The HEPES rinsing step before liposome introduction did not to affect the NPS signals of the liposome immobilization (Fig. 1b,c). Regarding the sonication time, two different times were studied, *i*.*e*., 20 min and 40 min. There was no remarkable difference in the peak shifts of the immobilized liposomes prepared by sonication for either 20 min or 40 min, thus, we decided to continue with the 20 min sonication method in the following liposome studies. Next, we immobilized DMPC, DSPC, and DLPC on the SiO_2_ sensor surface by using the same pretreatment with HNO_3_.

**Figure 2:**
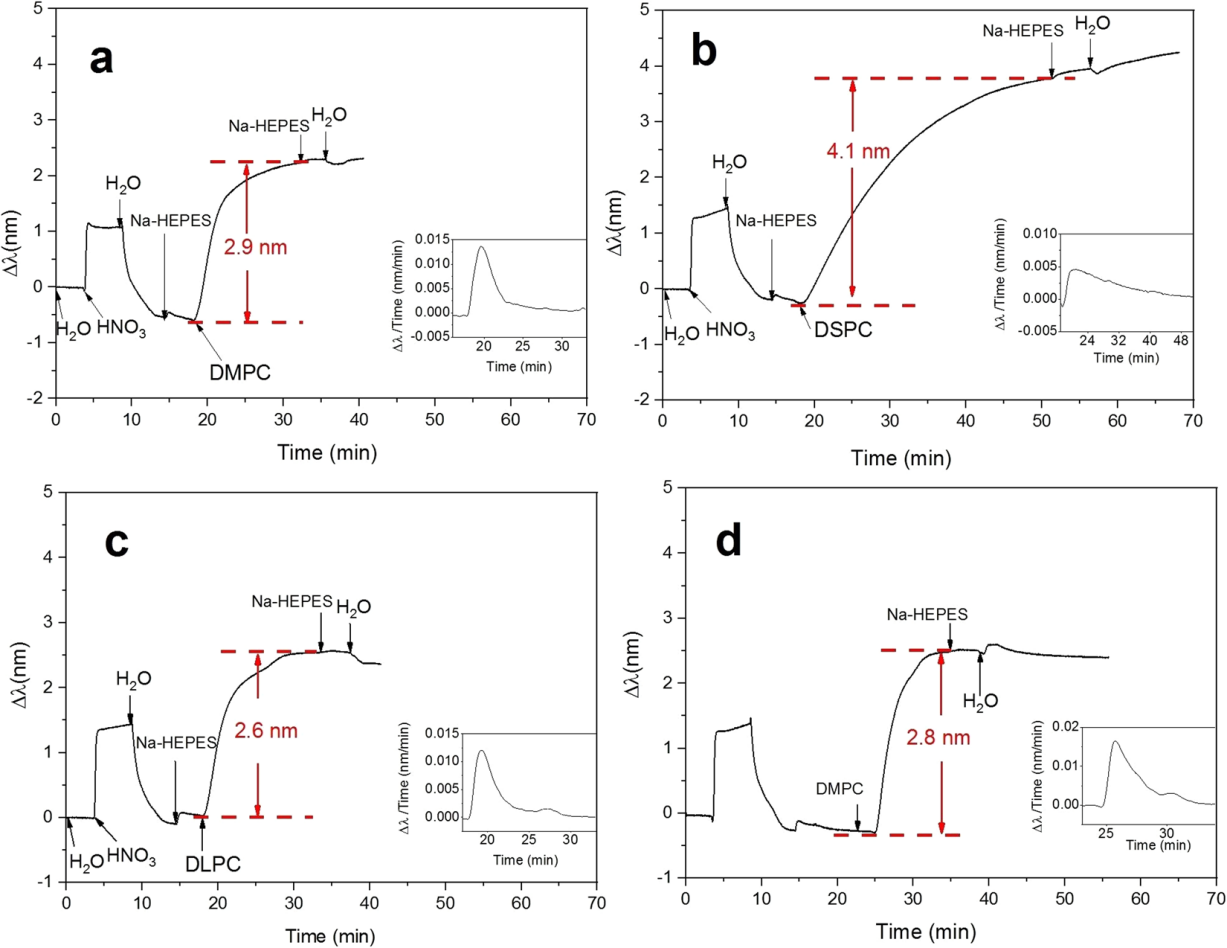
Immobilization of liposomes at different temperatures. (**a**) DMPC at 20 °C, (**b**) DSPC at 25 °C, (**c**) DLPC at 25 °C, and (**d**) DMPC at 35 °C. The insets of figures demonstrate the peak shift time derivative curves.

The DMPC liposomes were prepared by sonication for 20 min, and the sizes of the liposomes were 46 ± 15 nm (Table 1). Table 1 shows smaller liposome diameters for DMPC and DLPC. The probable reason is that the bending modulus for gel phase liposomes (DPPC and DSPC) at room temperature storage, are one order higher than those for fluid phase DLPC and DMPC at the same temperature^15,53^. The T_m_ of DMPC is 24 °C and to keep the temperature below the T_m_ the immobilization was done at 20 °C. The introduction of DMPC liposomes onto the sensor surface generated an increase in the peak shift (2.9 nm) (Fig. 2a). The subsequent HEPES buffer and water rinse did not induce a drop in the NPS signal, implying that the DMPC liposomes were strongly immobilized onto the SiO_2_ sensor. The peak shift time derivative curve (inset of Fig. 2a) demonstrates that the immobilization rate of DMPC liposomes (0.015 nm/min) was faster than that of DPPC liposomes (25 min), resulting in a shorter immobilization time for DMPC (15 min). This was probably due to the smaller size of the DMPC liposomes (46 ± 15 nm) as compared to the DPPC liposomes (above 70 nm). DMPC liposomes followed the immobilization pattern of DPPC liposomes and formed a SVL.

The sizes of the DSPC liposomes were 75 ± 7 nm after sonication for 20 min (Table 1). The T_m_ for DSPC is 55 °C. The immobilization procedure was conducted at 25 °C, hence, DSPC liposomes in the gel phase were immobilized. After the introduction of DSPC liposomes to the measurement chamber, an increase in the peak shift was observed (4.1 nm). A small continuous increase in the peak shift was still recorded after rinsing with HEPES buffer and water, implying that the DSPC liposomes were immobilized stably onto the SiO_2_ sensor surface, however, the sensor surface was not fully saturated yet. Based on the peak shift time derivative curve (inset of Fig. 2b), we could conclude that the DSPC liposomes were immobilized on the SiO_2_ sensor as a SVL. In addition, we noticed that the immobilization rate of DSPC liposomes (0.005 nm/min) was lower than that of DPPC liposomes, hence a longer immobilization time (30 min) was employed for DSPC liposomes.

The size of DLPC liposomes after 20 min sonication was 32 ± 5 nm (Table 1). The immobilization of DLPC liposomes was conducted at 25 °C, and since the T_m_ of DLPC is −2 °C, the DLPC liposomes were immobilized in the fluid phase. The introduction of DLPC liposomes increased the peak shift value (2.6 nm) (Fig. 2c). After the buffer (HEPES) and water rinse, there was no significant drop in the peak shift, implying that DLPC liposomes were strongly immobilized onto sensor. However, the curve during DLPC immobilization was different from that observed for the other liposomes. We noticed that there were two peaks in the peak shift time derivative of DLPC immobilization (inset of Fig. 2c). This suggests that the adsorption rate of DLPC onto the SiO_2_ sensor includes two different adsorption rates during the immobilization procedure. The reason for this unique pattern observed for DLPC liposomes can be related to the fluid phase immobilization of DLPC. The other three liposomes were immobilized in their gel states, and no such pattern was observed. To prove this, we studied the immobilization of another lipid, DMPC, with a T_m_ of 24 °C. When the immobilization temperature of DMPC was increased to 35 °C, *i*.*e*., above its T_m_, a similar pattern to DLPC adsorption appeared (Fig. 2d). The second peak in the peak shift time derivative indicated that there was another process apart from adsorption which increased the peak shift (inset of Fig. 2d). From the kinetics of the immobilization it could be speculated, that an uneven NPS sensor surface (*i*.*e*., the edge of the nanodiscs), which was less thermodynamically favorable for adsorption, produced the second lower rate of adsorption. A similar hypothesis was suggested by Jackman *et al*. for 160 nm 1-palmitoyl-2-oleoyl-*sn*-glycero-3-phosphocholine vesicles^51^.

### Transition temperature of phospholipids determined by NPS

**Figure 3:**
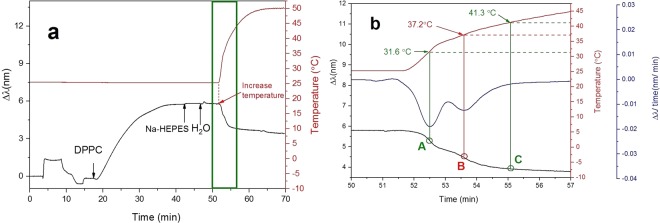
Phase transition of DPPC liposomes by NPS. The liposomes were immobilized at 25 °C and the peak shifts were recorded using a temperature gradient from 25 to 50 °C. (**a**) The peak shift and temperature profile of the whole measurement were recorded. (**b**) The peak shift, peak shift time derivative, and temperature profile between 50 and 57 min are shown.

To investigate the potential application of NPS in determining the T_m_ of phospholipids, we first immobilized DPPC liposomes (T_m_ of 41 °C) at 25 °C. At this temperature, DPPC liposomes are in the gel phase. After immobilization of DPPC, the sensor surface was rinsed with HEPES buffer and water. The peak shift was constant after the rinse, indicating the formation of a stable SVL of DPPC liposomes. Next, we started to increase the temperature of the measurement chamber from 25 °C to 50 °C. The temperature profile was recorded at the same time as the peak shifts (Fig. 3a). We observed that the peak shift dropped immediately when the temperature of the measurement chamber was increased. After approximately 3 min of temperature increase, the NPS signals leveled out. In order to investigate the change in the peak shift in more detail during the temperature increase, the NPS signals recorded from 50 to 57 min were further analyzed by calculating the time derivative of the peak shift value (Fig. 3b).

From the peak shift time derivative curve we noticed that two peaks appeared at 31.6 °C and 37.2 °C, indicating an acceleration decrease in the peak shift at 31.6 °C (point A in Fig. 3b) and 37.2 °C (point B in Fig. 3b). It was recently shown by Oh *et al*., that the kinetics of adsorption of SUVs are different based on the gel or fluid phase of the membrane^15^. However, there are no reports so far on changes in the NPS signal response due to a change in the membrane phase of already adsorbed SVLs. The membrane structure in the liquid crystalline phase is looser than in the gel phase because the disordered hydrocarbon chains of the phospholipid occupy more space. In addition, it has been reported that DPPC liposomes increase in size upon transition from gel to fluid phase^15^. The observed peak shift drop is in agreement with the published literature, where the described effect of temperature is not direct but the temperature affects more parameters having a total influence on the peak shift change (*i*.*e*., refractive index change of bulk solution and membrane, diameter of DPPC liposomes, deformation of liposomes, and vesicle substrate interaction)^15^. Some of those parameters change abruptly upon a transition from gel to fluid phase, which would in total produce an abrupt change of the peak shift at the point of phase transition. Therefore, the fast drop at 31.6 °C and 37.2 °C could be an indication of starting points for pretransition and main phase transition in the phospholipid membrane.

It is well known, that there are several phase transitions in DPPC^54^. Studies by DSC have shown, that the temperatures of the pretransition and the main phase transition of SUVs of DPPC are 28.0 °C and 37.2 °C, respectively, whereas the corresponding temperatures for large unilamellar vesicles (LUVs) of DPPC are 33.8 °C and 41.4 °C, respectively^18^. The T_m_ value of DPPC determined by DSC decrease as the diameter of the vesicles become smaller^17,18,55,56^. In our study, the size distribution peak (by number) of the DPPC liposomes prepared by sonication was wider than that of those prepared by extrusion. This implies that the DPPC liposomes in this study included also a small portion of LUVs. At 41.3 °C (point C in Fig. 3b), the peak shift leveled out, indicating that the conformational change of liposomes due to a phase transition was complete and the DPPC liposome phase changed to fluid state. In order to investigate the reversibility and possible hysteresis effect of the T_m_ measurement, multiple temperature cycles were conducted by NPS using DPPC liposomes. However, we found that the NPS signal was not stable when a frequent temperature change was applied to the system. Microbubbles accumulated in the fluidic system when the solution was heated above the solubility threshold, and this could not be avoided due to limitations in the instrumental setup (see Supplementary Information: Temperature cycling in NPS measurements using DPPC).

In the experiment above, the phase transition was investigated using a stable SVL of DPPC liposomes. In the following experiments, we investigated the phase transition of DPPC liposomes during their immobilization. The immobilization of sonicated DPPC liposomes took approximately 25 min and the temperature increase from 25 °C to 50 °C took 11 min. Hence, we first introduced DPPC liposomes into the measurement chamber for 4 min to obtain an increasing peak shift value, indicating that DPPC liposomes were adsorbing onto the sensor surface. After 4 min of DPPC liposome introduction, the temperature inside the measurement chamber was set to 50 °C, while the liposomes were continuously introduced into the system. Both the peak shift and the temperature profile were recorded during the whole immobilization procedure.

**Figure 4:**
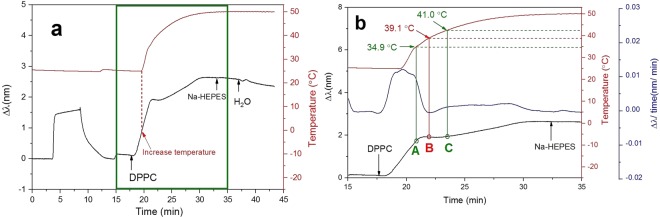
Detection of phase transition of DPPC liposomes by NPS. Liposome immobilization was done during a continuous increase in the temperature from 25 °C to 50 °C. (**a**) The peak shift and temperature profile of the whole measurement. (**b**) The peak shift, peak shift time derivative, and temperature profile between 15 and 35 min.

A new pattern of peak shift was obtained for liposome adsorption (Fig. 4a). For the DPPC liposomes adsorbed at a constant temperature of 25 °C, the peak shift generally increased during the whole adsorption procedure (Fig. 1). However, when the DPPC liposomes were immobilized simultaneously as the temperature was increased, a small drop in the peak shift was observed. This was followed by a continuous increase in the peak shift, until reaching a stable peak shift at the end. To further demonstrate the change in the peak shift during an increase of the measurement chamber temperature, the NPS signals obtained from 15 to 35 min were further analyzed by taking the time derivative of the peak shift values (Fig. 4b).

At 34.9 °C (point A in Fig. 4b), the rate of the increase in the peak shift started to drop rapidly. There are two possible explanations for this. One is that the sensor surface was almost saturated with liposomes. However, when the temperature was further increased, there was a continuous increase in the peak shift. Hence, the slow increase rate of the peak shift at 34.9 °C was probably not due to saturation of the sensor surface. Another possible explanation was that there was a conformational change of DPPC liposomes at 34.9 °C. This could be an indication of a pretransition state. As the temperature kept increasing, the peak shift time derivative dropped below zero, showing the strongest temperature effect at 39.1 °C (point B in Fig. 4b). At this temperature, the peak shift time derivative dropped to its lowest value (−0.002 nm/min). When the temperature was further increased to 41.0 °C (point C in Fig. 4b), the peak shift began to increase steadily and reached a stable level at 30 min.

Based on the results above, we assume that the liposome gel structure started to disorganize at 34.9 °C during the immobilizing step. This would indicate a start of the lipid phase pre-transition. At 39.1 °C, the peak shift seemed to be predominantly influenced by the change of the liposomal structure, suggesting the start of the main phase transition. As the temperature continued to go up, a slow increase of the peak shift was observed. An acceleration of the peak shift change was observed again at 41.0 °C suggesting that the transition to the fluid structure was finished and the only observed effect was liposome adsorption. This result was very similar to the end point temperature of the phase transition for fully immobilized SVLs of DPPC (41.3 °C, see Fig. 3). Furthermore, this value determined by NPS (41.3 °C or 41.0 °C) was very close to the T_m_ determined by DSC (41.4 °C)^18^. Even though the methods are based on the analysis of different physical properties, the obtained T_m_s are highly comparable.

Based on the presented data, our explanation of the observed effect would be that gel phase liposomes adsorb with a slight deformation at the surface of the sensor. Opposite to vesicles supported on TiO_2_, increasing temperature induces DPPC liposome deformation to cover more SiO_2_ surface^15^. This is opposite to what has been observe with DPPC vesicles on TiO_2_ surface. As they spread over a surface they occupy free sensing surface and, therefore, decrease the adsorption rate. At the point where the system passes the phase transition temperature this decrease is most obvious (demonstrated by the lowest peak shift time derivative, see point B in Fig. 4b), which suggests considerably higher deformation of liposomes entering a liquid crystalline phase. After this point the adsorption became more pronounced and the peak shift started to increase again until reaching a surface fully covered with SVL. For a better interpretation of this effect, a schematic illustration (Fig. S7) of the relation between the change in the NPS signal and vesicle deformation during immobilization with phase transition was produced (see Supplementary Information Fig. S7).

To confirm that the new pattern of NPS signals was not merely due to a temperature effect, we conducted a reference run using DSPC. The T_m_ for DSPC is 55 °C meaning that during the temperature increase from 25 °C to 42 °C, the DSPC liposomes remained in the gel phase. The same protocol as for DPPC analysis was used for DSPC. Both the temperature profile and the peak shift were recorded, and they are shown together with the peak shift time derivative in Fig. S2. This confirms that NPS has the potential of being a valuable method for the determination of the phase transition of phospholipids.

**Figure 5:**
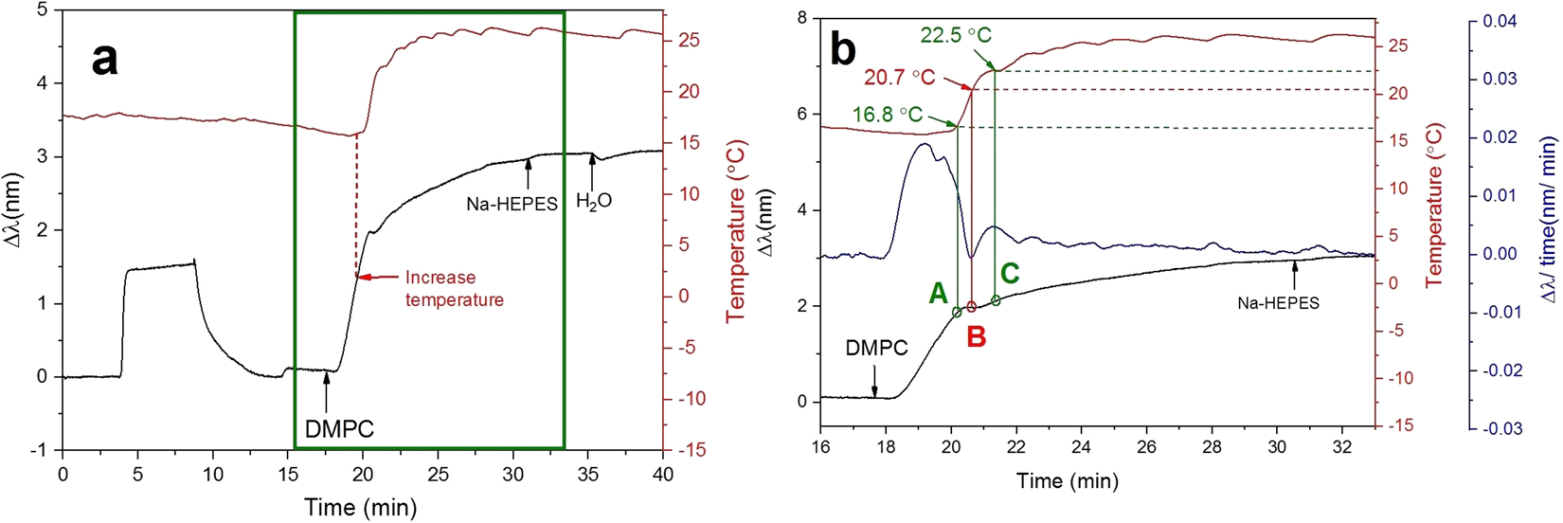
Detection of DMPC liposomes phase transition during the immobilization procedure. Liposome immobilization was done at 16 °C. The studied temperature range was 16 °C to 26 °C. (**a**) The peak shift and the temperature profile of the whole measurement were recorded. (**b**) The peak shift, peak shift time derivative, and temperature profile between 16 and 33 min are shown.

The ability of NPS to analyze T_m_ was further investigated using DMPC and DSPC liposomes with T_m_s of 24 °C and 55 °C, respectively^57^. The liposomes composed of DMPC were first immobilized in the gel state at 16 °C, and a steady increase in the peak shift was observed. After 4 min of immobilization, the temperature of the measurement chamber was increased from 16 °C to 26 °C. At the beginning of the temperature increase, the peak shift still increased, however, at a certain point, a small rapid drop in the peak shift occurred. After that, the peak shift continued to increase until reaching a plateau (Fig. 5a). The time derivative of the peak shift between 16 and 33 min is shown in Fig. 5b together with the peak shift and the temperature profile. At around 16.8 °C, a fast decline of the peak shift time derivative was observed, indicating a slow increase in the peak shift (point A in Fig. 5b). Upon a further increase in the temperature, the peak shift stopped increasing and instead began to drop. At 20.7 °C, the lowest value of the peak shift time derivative (−0.001 nm/min) was observed, *i*.*e*., the fastest drop in the peak shift happened at this temperature (point B in Fig. 5b). After that, the decrease in the peak shift slowed down and started to increase again. We noticed that the time period of the drop in the peak shift was smaller for DMPC than for DPPC. This can be related to the hydrocarbon chain length. The hydrocarbon chains in DPPC and DMPC are C16 and C14, respectively. It could also be due to the faster temperature increase during the DMPC liposomes immobilization. When the temperature was higher than 22.5 °C, there was an increase in the peak shift (point C in Fig. 5b). According to the analysis of the peak shift time derivative curve, we assume that the pretransition of DMPC started at 16.8 °C. This was close to the values measured in previous studies^57,58^. The main phase transition determined by NPS started at 20.7 °C and ended at 22.5 °C. This end value is similar to the transition temperature (23 °C) determined by DSC^57^. A reference run was conducted using DPPC liposomes (Fig. S3), which further confirms that the drop in the NPS signals during DMPC immobilizing was due to a phase transition and not due to a viscosity change because of the temperature gradient.

**Figure 6:**
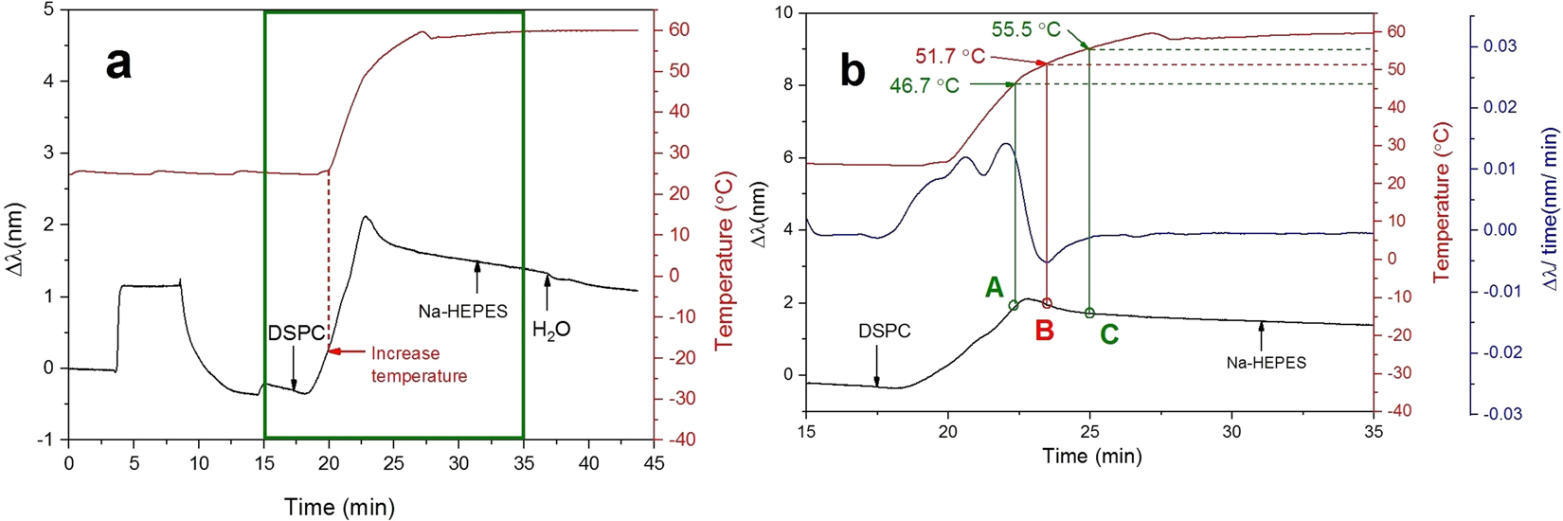
Immobilization of DSPC liposomes using a temperature gradient from 25 °C to 60 °C. (**a**) The peak shift and temperature profile of the whole measurement were recorded. (**b**) The peak shift time derivative and temperature profile between 15 and 35 min are shown.

To study the effect of using a temperature gradient on immobilization of DSPC liposomes, the liposomes were first immobilized onto the sensor at 25 °C. At this temperature, the liposomes were in the gel phase. An increase in the peak shift was observed and after 4 min of liposome immobilization, the temperature in the measurement chamber was increased from 25 °C to 60 °C. When the temperature approached the T_m_ of the lipid, the peak shift started to drop (Fig. 6a). The same change in the signal was observed for DPPC and DMPC as well. To further investigate the NPS signals of DSPC immobilization during the increasing temperature gradient, the peak shift time derivatives from 15 to 35 min were calculated (Fig. 6b). Starting from 46.7 °C (point A in Fig. 6b), the increase in the peak shift slowed down significantly. At 51.7 °C (point B in Fig. 6b), the peak shift rapidly dropped with a rate of −0.005 nm/min. After that the peak shift time derivative started to increase again and when the temperature was above 55.5 °C (point C in Fig. 6b), the peak shift time derivative remained stable but slightly negative. This indicates that the peak shift kept dropping linearly at a very slow rate.

The peak shift pattern of DSPC was slightly different from those recorded for DPPC and DMPC. In the case of DPPC and DMPC, the peak shift started to increase after the small drop. The reason for this is probably related to the absolute reached temperature, because the absolute peak shift of the immobilization got gradually smaller when moving from liposomes with lower T_m_ to higher values (see Figures in order from 5, to 4 and 6). Based on the analysis of the peak shift time derivative curve, we assume that the pretransition of DSPC started at 46.7 °C, which is very close to the values shown in previous study^59,60^. The main phase transition started at 51.7 °C and ended at 55.5 °C (Fig. 6). The transition temperature of DSPC determined by DSC was inside this range^57,60^. The liposome temperature transitions were determined by QCM for the comparison with NPS (See Supplementary Information: QCM analysis of SVL temperature transitions).

### Interaction of an ionic liquid [P_14444_][OAc] with liposomes in gel and fluid states

In order to explore the potential application of NPS in investigating phase transition properties of liposomes, the interaction of a model compound, [P_14444_][OAc] ionic liquid (IL), with liposomes in different phases was investigated. The IL was selected based on previous results using other methodologies^50^. There was no strong electrostatic or hydrophobic interactions between [P_14444_][OAc] and the SiO_2_ surface (Fig. S8).

**Figure 7:**
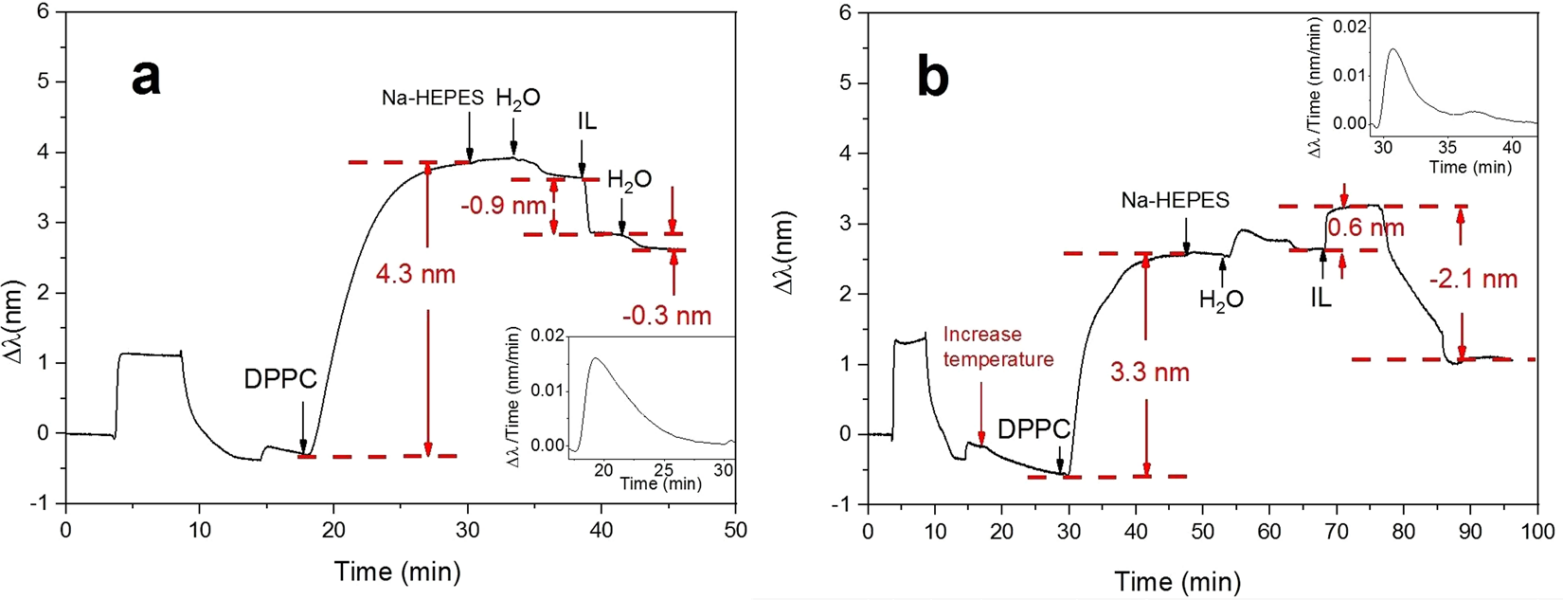
Interactions between DPPC liposomes and [P_14444_][OAc] by NPS. (**a**) Gel phase DPPC liposomes. (**b**) Fluid phase DPPC liposomes. The insets of figures demonstrates the peak shift time derivative curve.

After HNO_3_ pretreatment, gel phase DPPC liposomes were immobilized onto the SiO_2_ sensor surface at 25 °C. The peak shift increased to 4.3 nm, and after rinsing with HEPES buffer and water for a few minutes, the peak shift remained constant, indicating that a stable SVL of DPPC liposomes was formed on the sensor (Fig. 7a). [P_14444_][OAc] (1 mM) was introduced to the system, and an immediate drop of the peak shift (−0.9 nm) was observed. Part of the phospholipids probably form IL-phospholipid aggregates and these were flushed away from the sensor surface^61,62^. The subsequent water rinse further lowered the peak shift (−0.3 nm), suggesting that more phospholipids from the SVL of DPPC liposomes were removed from the sensor surface.

Next, DPPC liposomes were immobilized at 50 °C onto SiO_2_ (Fig. 7b). Immobilization of fluid phase DPPC liposomes lead to a 3.3 nm increase in the peak shift, which was smaller than that at 25 °C (4.3 mm). These results were consistent with previous research^15^. It has been shown that the DPPC liposomes in the gel phase are packed more closely than DOPC liposomes in the fluid phase^34^. Hence, we assume that the higher peak shift of DPPC in gel phase was related to the more densely packed layers. To confirm that the difference in the peak shift was mainly due to phase transition instead of the effect of temperature, DLPC liposomes were immobilized at 25 °C and 50 °C. Similar increase of peak shift was obtained (See Supplementary Information Fig. S9). In addition, we noticed that there were two peaks in the peak shift time derivative when immobilizing DPPC at 50 °C (inset of Fig. 7b). This phenomenon also happened when immobilizing DLPC (Fig. 2c) and DMPC (Fig. 2d) in their fluid phase. We believe that this NPS signal pattern during liposome immobilization is due to the particular characteristics of vesicles in the fluid phase. A previous research has demonstrated that a SLB was formed when DPPC was immobilized on SiO_2_ at 50 °C^15^. It was shown that there was a clear rate acceleration during the liposomes immobilization, which indicated the formation of SLB. However, in our study a significant rate acceleration was not observed. The peak shift time derivative curve suggests that we did not obtain a SLB of DPPC liposomes. In addition, the rupture of liposomes at a defined temperature would be followed by a sharp increase in the frequency detected by QCM. However, in our QCM results (see Supplementary Information) no sharp increase in the frequency was observed. Hence, based on our NPS and QCM results, the rupture of liposomes could not be confirmed, which might be related to the different pretreatments and buffer solution used in our study.

After obtaining a stable SVL of DPPC liposomes, [P_14444_][OAc] was introduced and an increase in the peak shift was recorded (0.6 nm). This was opposite to the behavior of gel phase DPPC. Since the DPPC liposomes in fluid phase were not as rigid as in the gel phase, it is possible that some [P_14444_][OAc] molecules were capable of entering the bilayer. The deformation of liposomes resulted in an increase in the peak shift. A subsequent water rinse resulted in a drop in the peak shift (−2.1 nm), indicating that a considerable part of DPPC liposomes was removed from the sensor surface. The drop in the peak shift of DPPC in the fluid phase was larger than that in the gel phase. Therefore, DPPC in the fluid phase seemed to be more sensitive and less resistant to [P_14444_][OAc] than DPPC in the gel phase. This is well in accordance with previous studies demonstrating that gel phase liposomes are less prone to interactions with external additives^63–65^.

**Figure 8:**
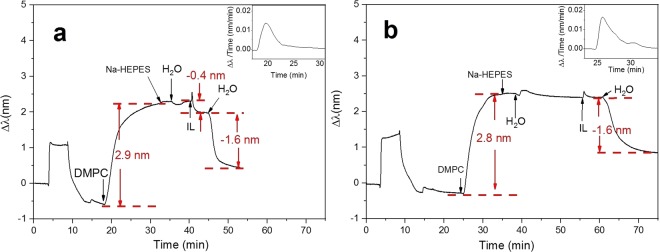
Interactions between DMPC liposomes and [P_14444_][OAc]. (**a**) DMPC liposomes in gel phase. (**b**) DMPC liposomes in fluid phase. The insets of figures demonstrates the peak shift time derivative curve.

In addition to DPPC liposomes, DMPC liposomes (T_m_ of 24 °C) in the gel and fluid phase were also treated with 1 mM [P_14444_][OAc] at 20 °C and 35 °C (Fig. 8a,b). In the gel phase of DMPC liposomes, the introduction of [P_14444_][OAc] lead to a spike in the peak shift, followed by a drop in the value (−0.4 nm). The fast initial increase was due to the partitioning of [P_14444_][OAc] into the liposomes, which further deformed the liposomes resulting in a peak shift drop of −0.4 nm. A subsequent water rinse further decreased the peak shift (−1.6 nm), implying that the IL-liposome mixed system was partly flushed away from the sensor surface. With the fluid phase DMPC liposomes, the introduction of [P_14444_][OAc] did not result in a drop in the peak shift. The signal first increased then dropped slightly, but the peak shift was not lower than before introducing [P_14444_][OAc]. This change in the signal implies that the partitioning of [P_14444_][OAc] did not make the structure more loose, since the fluid phase DMPC was already in a loose conformation. A subsequent water rinse decreased the peak shift (−1.6 nm), indicating that part of the liposomes was removed from the sensor surface.

## Summary

In this study, we have demonstrated that NPS can be used for investigating the conformational change of liposomes when they undergo phase transition. During the liposome immobilization procedures, the increase in the NPS signals slowed down significantly when the system temperature got close to the T_m_. A drop in the peak shift was observed as the system temperature kept increasing and approached the T_m_. While there are works analyzing the temperature effect on fully immobilized SVLs, this is, to the best of our knowledge, the first study showing that NPS can be used for determining the T_m_ of phospholipids directly during vesicle immobilization. Furthermore, in the interaction of [P_14444_][OAc] with gel and fluid phase liposomes, our results show that NPS is able to differentiate the effects between [P_14444_][OAc] and gel and fluid phase liposomes. Future studies will be required to overcome the limitations of temperature cycling using the NPS instrument. To conclude, NPS appears as a novel and promising technique in investigating the properties of biomembrane phase transition of adsorbed liposomes.

## Methods

### Solution preparation

HEPES (Sigma, Darmstadt, Germany) buffer solution with an ionic strength of 10 mM and a pH value adjusted to 7.4 ± 0.05 by sodium hydroxide (J.T. Baker Chemicals, Center Valley, PA, USA) was used throughout the study. The HEPES buffer solution was filtered through a 0.45 µm filter (Gelman Sciences, Ann Arbor, MI, USA) before use. The ionic liquid [P_14444_][OAc] was synthesized at the Department of Chemistry at the University of Helsinki, Finland^66^ and diluted with MilliQ water to 1 mM solution.

### Liposome preparation

Phospholipids DPPC (850355C), DMPC (850345C), DSPC (850365C), and 1,2-dilauroyl-*sn*-glycero-3-phosphocholine (DLPC, 850335C) in chloroform were purchased from Avanti Polar Lipids (Alabaster, AL, USA). The liposomes of DPPC were prepared by extrusion and sonication. The rest of the liposomes were prepared by sonication using a sonication bath. Please see the Supplementary Information for details on the preparation of the liposomes (Supplementary Information: Liposome preparation).

### Nanoplasmonic sensing measurements

The NPS instrument was an Insplorion XNano II instrument (Insplorion AB, Gothenburg, Sweden). The instrument setup has previously been described in detail^50^. SiO_2_ coated sensors were purchased from Insplorion (Insplorion AB, Gothenburg, Sweden). Details on sensor pretreatment and liposome immobilization can be found in Supplementary Information: Nanoplasmonic sensing (NPS) sensor pretreatment and liposome immobilization. To investigate the main phase transition of liposomes, the temperature inside the measurement chamber was adjusted to appropriate values after or during the immobilization of the liposomes. The temperature profile was recorded at the same time with the NPS signals (peak shift). We anticipate that the heating of the glass sensor will be delayed compared to the steel cell, however the flowing liquid will heat up in the steel input channel, and subsequently it will heat up the surface with the adsorbed liposomes. Therefore, the actual sensor surface temperature will be very close to the measured one. The reference signal of 1 mM [P_14444_][OAc] was recorded before its interaction with DPPC and DMPC liposomes. Interactions between [P_14444_][OAc] and liposomes were conducted by introducing [P_14444_][OAc] into the measurement chamber after stable liposome immobilization on the sensor surface. All the NPS measurements were conducted twice. Quartz crystal microbalance (QCM) measurements are in Supplementary Information.

## Electronic supplementary material

Supplementary Information

## Data Availability

All data generated or analyzed during this study are included in this published article (and its Supplementary Information files).
