# Supplementary material for: Determination of the Main Phase Transition Temperature of Phospholipids by Nanoplasmonic Sensing
Source: Sci Rep. 2018 Oct 4;8:14815. doi: 10.1038/s41598-018-33107-5 (PMC6172256; doi:10.1038/s41598-018-33107-5)
Supplement: Supplementary file 1 — Supplementary Information [file 41598_2018_33107_MOESM1_ESM.docx]

Supplementary Information

Determination of the Main Phase Transition Temperature of Phospholipids by Nanoplasmonic Sensing

Wen Chen, Filip Duša, Joanna Witos, Suvi-Katriina Ruokonen, Susanne K. Wiedmer*

**Temperature cycling in NPS measurements using DPPC.** DPPC liposomes were first immoblized onto the SiO_2_ sensor. The temperature cycling was conducted in two ways: without water flow (Figure S1a) and with water flow (Figure S1b). Without water flow, we observed that the NPS signals repeated as the temperature finished one cycle (25°C - 50°C - 25°C). However, after three cycles of temperature change, the extinction increased dramatically. The normal level for extinction is around 0.5 to 0.6. The high level of extinction (above 0.75) indicated scattering of light, probably caused by expanding air bubbles. In the case of continuous water flow, a similar increase of extinction level was observed at the beginning of the second temperature cycle. The increase of extinction had an effect on the NPS signal. Based on these results, it seems that further studies on an upgraded system with a proper heating/cooling control device are required in order to avoid microbubble formation and accumulation during temperature cycling NPS measurements.

**
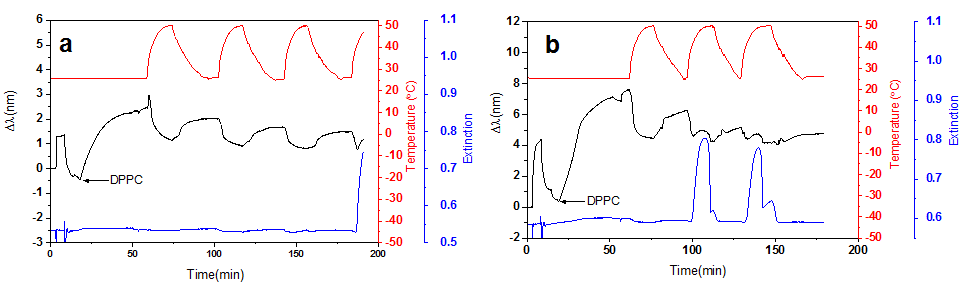
**

**Figure S1.** NPS measurements for DPPC with temperature cycling: (a) without flow and (b) with flow.


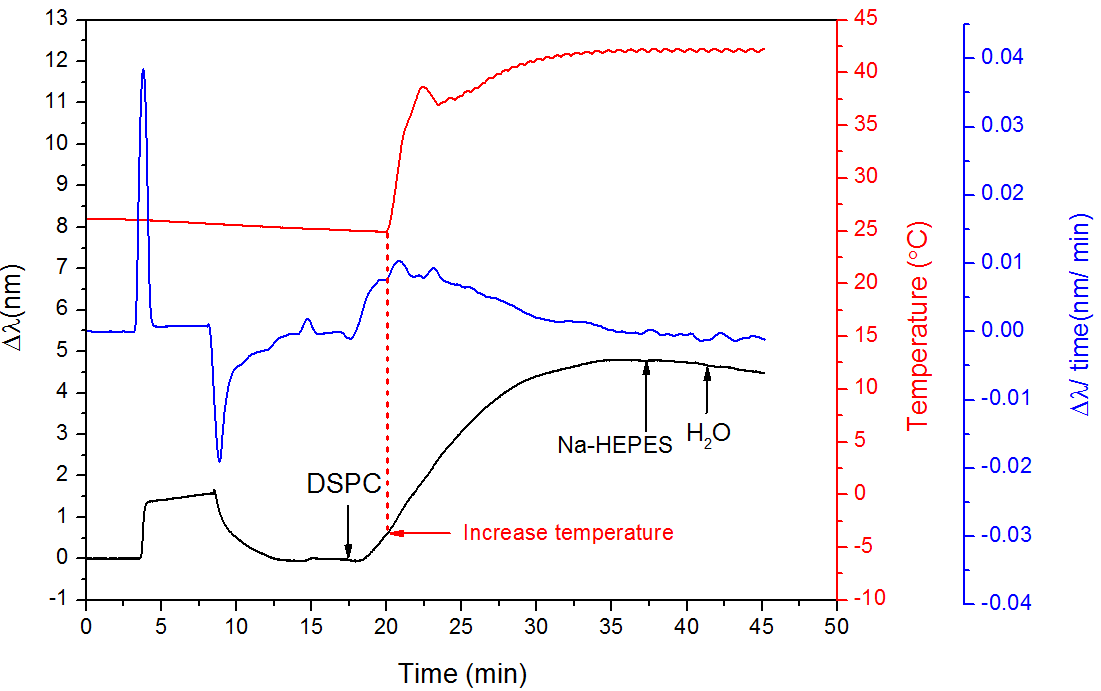


**Figure S2.** Immobilization of DSPC liposomes keeping the system below the phase transition temperature during the whole immobilization (temperature range from 25 °C to 42 °C). During the increase of the temperature, there was no drop in the peak shift. This is also seen from the peak shift time derivative, which shows that the peak shift increased fast at the beginning of DSPC immobilization. Later on, the increase of peak shift slightly slowed down, but the peak shift did not drop during the immobilization procedure.


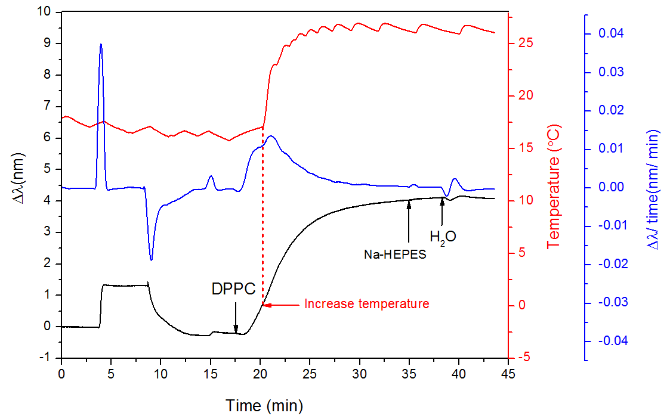


**Figure S3.** Immobilization of DPPC liposomes using a temperature gradient from 16 °C to 26 °C. DPPC was in the gel state during the whole procedure. No sudden change in the peak shift curve was observed. The DPPC liposomes were immobilized onto the SiO_2_ sensor surface during a temperature increase from 16 °C to 26 °C. In this temperature range, DPPC liposomes (T_m_=41.4 °C) are in the gel state. We observed that the peak shift increased without any drop in the signal during the whole DPPC immobilization procedure.

**Figure S4.** QCM analysis of DMPC liposome adsorption with continuous increase of the flow cell temperature. The full lines show the change in the frequency, while the dotted lines show the actual temperature of the cell. The flow rate during the whole analysis was 50 µL/min, with a maximum heating rate of 0.8 °C/min. The zero point of the x-axis means switching of the selector valve to liposome dispersion after pretreatment of the sensor, done as described in the Methods section.

**Figure S5.** QCM analysis of DPPC liposome adsorption with continuous increase of the flow cell temperature. The full lines show the change in the frequency, while the dotted lines show the actual temperature of the cell. The flow rate during the whole analysis was 50 µL/min, with a maximum heating rate of 0.8 °C/min. The zero point of the x-axis means switching of the selector valve to liposome dispersion after pretreatment of the sensor, done as described in the Methods section.

**Figure S6.** QCM analysis of DSPC liposome adsorption with continuous increase of the flow cell temperature. The full lines show the change in the frequency, while the dotted lines show the actual temperature of the cell. The flow rate during the whole analysis was 50 µL/min, with a maximum heating rate of 0.8 °C/min. The zero point of the x-axis means switching of the selector valve to liposome dispersion after pretreatment of the sensor, done as described in the Methods section.

**Schematic illustration of changes in the NPS signals.** During the immobilization procedure gel phase liposomes attach to a SiO_2_ sensor surface and deform slightly. This leads to an increase in the NPS signal (Figure S7). When the temperature increases to the T_m_, the vesicles deform into a biscuit‑resembling shape. The deformation at higher coverage level causes a sudden decrease of space available for the adsorption, which is seen by NPS as a decrease of the time derivative of peak shift (blue line in the inset). The rest of the available space on the sensor surface is then slowly filled with the fluid phase liposomes until full coverage is reached.


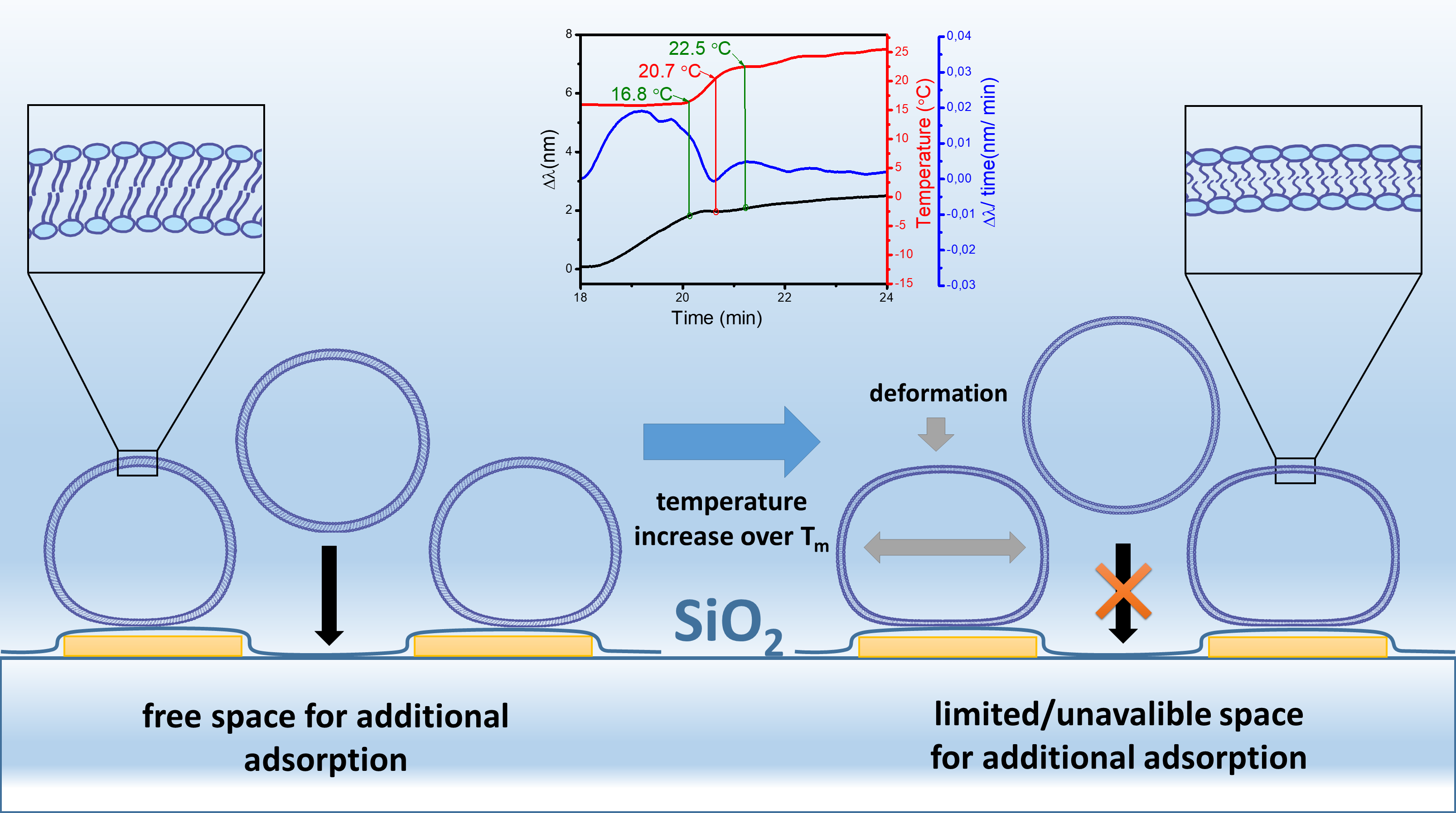


**Figure S7.** A schematic illustration of the relation between changes in the NPS signals and vesicle adsorption during phase transition.


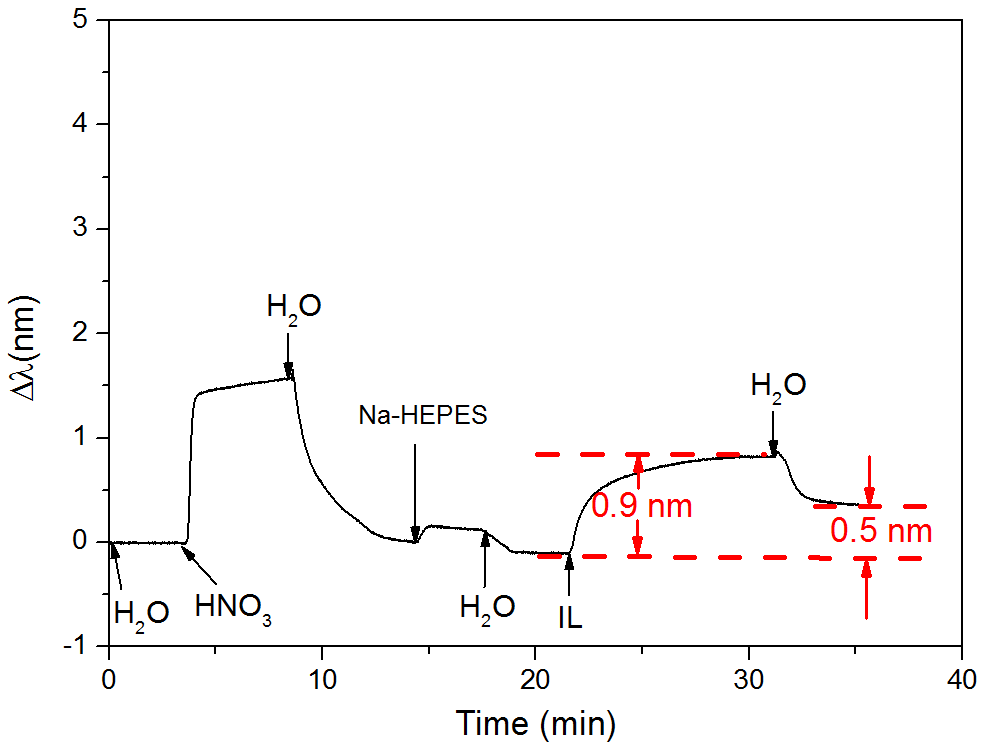


**Figure S8.** Reference (blank) values in NPS of [P_14444_][OAc] (marked as IL) on the SiO_2_ sensor. To get information about possible interactions between [P_14444_][OAc] and the SiO_2_ coated sensor we first investigated the reference (blank) signal of the immobilization. After introducing [P_14444_][OAc] to the system, an increase in the peak shift (0.9 nm) was observed. A following water rinse removed most of the IL from the surface. However, the total increase in the peak shift was not more than 0.5 nm, which indicates that the interaction between the IL and the sensor surface is weak.


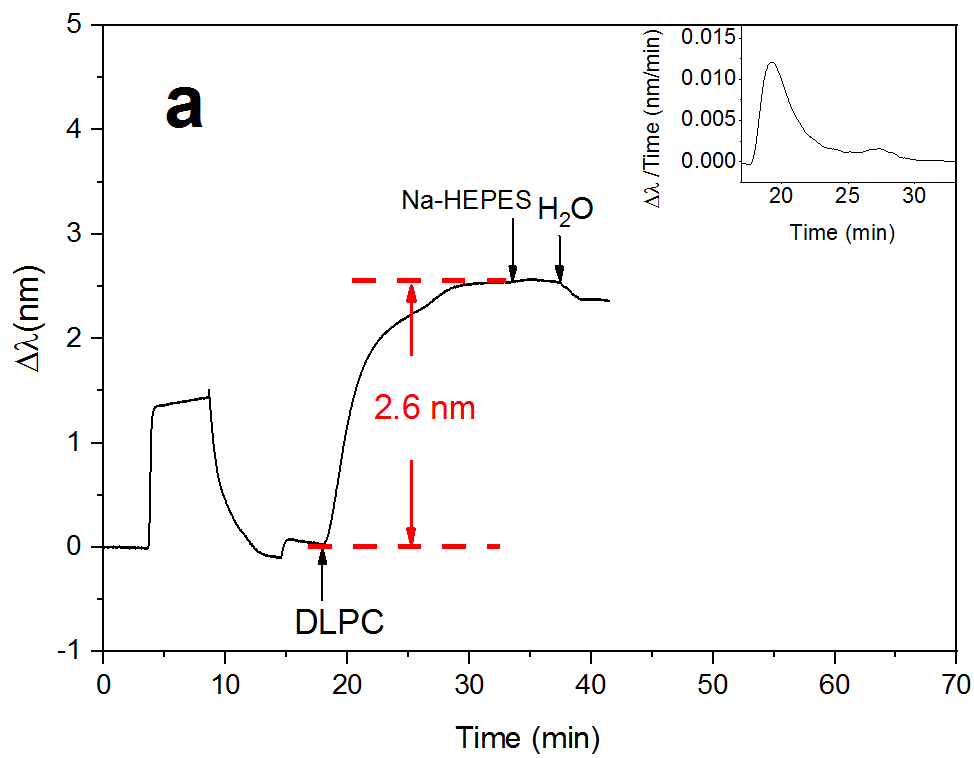

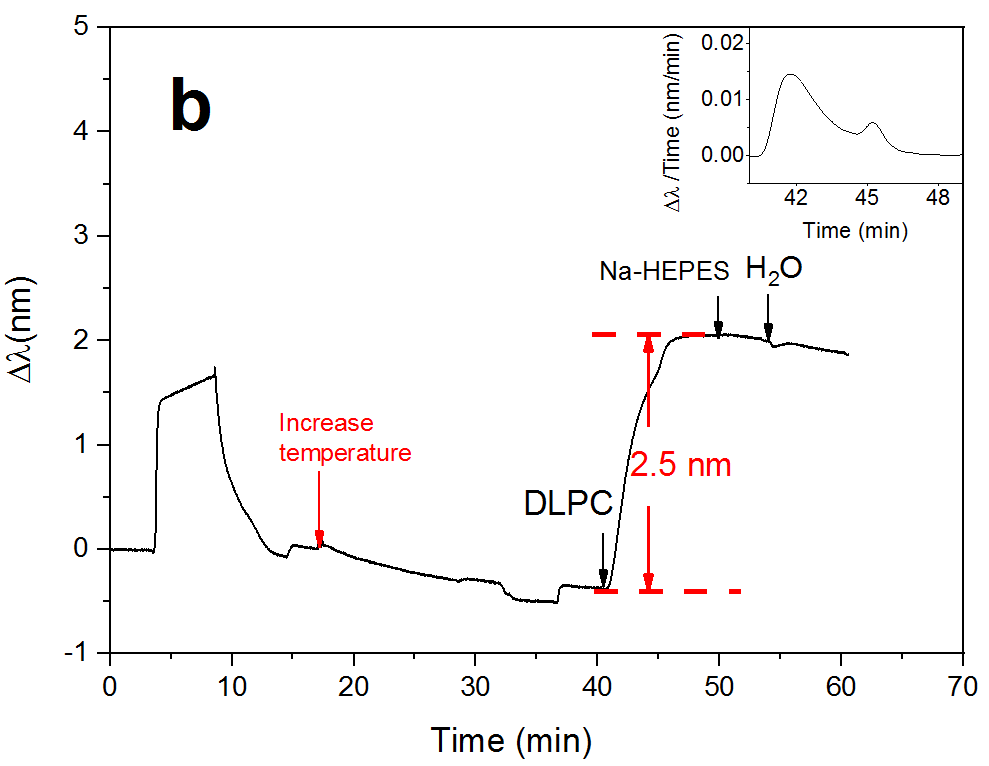


**Figure S9.** Immobilization of DLPC liposomes at (a) 25 °C and (b) 50 °C. Since, the T_m_ of DLPC is -2 °C, the DLPC liposomes were in the fluid phase at both temperatures. The results show a similar increase of peak shift; 2.6 nm and 2.5 nm at 25 °C and 50 °C, respectively. This indicates that the increase of peak shift during immobilization was not affected by the temperature.

**Liposome preparation.** For the preparation of the liposomes, chloroform was first evaporated under a gentle flow of air to form a dry film of lipids, and traces of chloroform were removed by desiccation under vacuum overnight. The lipid film was rehydrated in HEPES buffer solution. The solution was agitated (Biosan, TS-100 Thermo Shaker, Riga, Latvia) at a speed of 800 RPM at 70 °C for 1 hour to obtain a dispersion of multilamellar vesicles (MLVs). In the extrusion method, the dispersion of MLVs was extruded 19 times through Millipore 100 nm pore size polycarbonate filters (Bedford, MA, USA) by a Liposo-Fast extruder at a temperature of at least 10 °C above the T_m_ of the lipid. In the sonication method, the dispersion of MLVs was sonicated in a bath sonicator (Elmasonic P, Elma, Singen, Germany) at a frequency of 37 kHz (power of 100%) for 20 and 40 minutes. The temperature in the sonication bath was 65 °C. The liposomes were filtered through a 0.45 µm PVDF syringe filter (B. Braun, Inject-F, Melsungen, Hessen, Germany) before use. After filtration, the liposome dispersion was diluted with HEPES to a concentration of 0.11 mg/mL and kept at room temperature (22 °C). The parameters of agitation and sonication for different liposomes are summarized in Table S1. The size distributions of liposomes (by number) were determined by a Zetasizer Nano ZS instrument (Malvern Instruments, Malvern, Worcestershire, U.K.). One liposome dispersion was measured 7 times, and each measurement consisted of 12 to 15 repetitions.

**Table S1.** Parameters of agitation and sonication for the preparation of DPPC, DMPC, DSPC, and DLPC liposomes.

| **Phospholipids** | **Agitation temperature (°C)** | **Sonication** | |
| --- | --- | --- | --- |
|  |  | **Temperature (°C)** | **Time (min)** |
| DPPC | 70 | 65 | 20 and 40 |
| DMPC | 60 | 40 | 20 |
| DSPC | 70 | 65 | 20 |
| DLPC | 60 | 40 | 20 |

**Nanoplasmonic sensing (NPS) sensor pretreatment and liposome immobilization.** Before the first measurement, the SiO_2_ sensor was pretreated for 20 min with an UV ozone cleaner (UVC-1014 NanoBioAnalytics, Berlin, Germany). Between each measurement, the sensor was immersed in methanol and cleaned in a sonication bath for 5 minutes (sweeping mode, frequency of 80 kHz, and power of 30 %). After sonication, the sensor was dried under a gentle stream of compressed air. Three SiO_2_ sensor chips were used in this study. The quality of the used SiO_2_ sensor chips was confirmed by checking the bulk refractive index (RI) sensitivity values, which were 97.37, 106.47, and 104.03 nm per RI unit (Figure S10). To immobilize the liposomes, the sensor was pretreated as follows: 3 min with water, 5 min with HNO_3_ (2 M), 6 min with water, and 3 min with HEPES buffer at a flow rate of 100 µL/min. After the pretreatment, the liposomes were introduced to the system until a stable signal was obtained, followed by a flush with HEPES buffer and water.

**
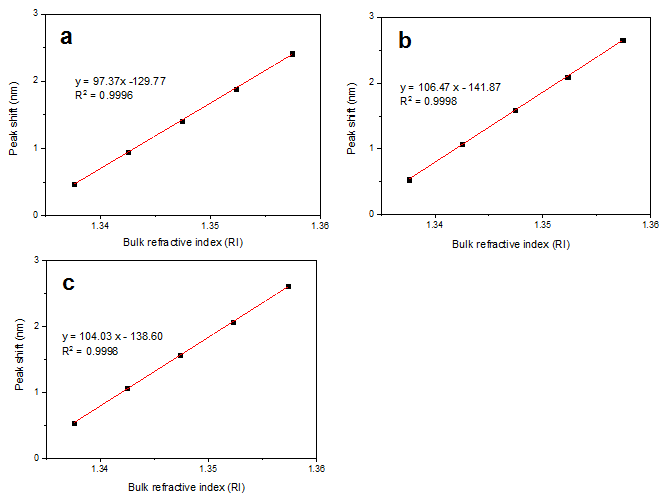
**

**Figure S10.** Bulk refractive index sensitivity in NPS of three silicon dioxide sensor chips.

**Quartz crystal microbalance (QCM) measurements.** 10 MHz quartz crystals (International crystal manufacturing, Oklahoma City, OK, USA) with golden electrodes coated with 5 nm of SiO_2_ were used for QCM analysis. A QCM unit with a polycarbonate flow cell (KEVA, Brno, Czech Republic) was used. The temperature was measured using an inserted temperature probe. A REGLO Digital peristaltic pump (Ismatec, Wertheim, Germany) maintained the flow rate at 50 µL/min and was interfaced via a RS232 port to the PC. The inlet side of the peristaltic pump tubing was connected to a 10-port selector valve (VICI AG International, Schenkon, Switzerland), which was operated by a Cole-Parmer Data Acquisition Module 18200-00 (Cole-Parmer, Vernon Hills, IL, USA). The whole fluidic setup and QCM data acquisition was further controlled by a custom designed Labview application (National Instruments, Austin, TX, USA). The temperature of the cell was controlled manually by increasing the starting temperature to 35°C, 55°C, and 60°C for DMPC, DPPC, and DSPC, respectively.

**QCM sensor pretreatment and liposome immobilization.** Before each measurement the sensor was sonicated for 5 min (37 kHz, 10% power) in chloroform to remove any possible contamination, briefly rinsed with methanol after the sonication, and dried with cleaned compressed air. The immobilization of liposomes was always preceded by a rinsing sequence composing of 5 min of water, 5 min of 2 M HNO_3_, 5 min of water, and 5 min of HEPES buffer. The solutions containing liposomes were applied directly after the 20 min pretreatment sequence. OriginPro 8.6 software (OriginLab, Northampton, MA, USA) was used for evaluation of the acquired data.

**QCM analysis of SVL temperature transitions.** QCM with impedance monitoring was utilized as a complementary surface sensing technique to further analyze the abrupt changes in the signal at the phase transition temperature region. So far there has been a handful of works on T_m_ analysis [1-3], but in all cases the actual detection of T_m_ has been indirect, following the completed analysis. Therefore, we mirrored the NPS analysis method with QCM to check whether we are able to observe a similar kink in the signal. Blank analysis with HEPES buffer rinse instead of liposome dispersion was done to evaluate the temperature effect on the QCM sensor itself.

In all the heating steps, we observed an increase in the frequency upon heating of the flow cell (Figures S4, S5, and S6). The increase of the frequency caused by the temperature gradient could not be compensated by a decrease in the frequency caused by continuing adsorption of liposomes. In addition, the temperature effect on the sensor exhibited wavy discrepancies, especially in the DPPC and DSPC experiments. This was most probably due to deformation of the polycarbonate flow cell material. Moreover, as the heating was done using a fan blowing tempered air from a Peltier element, the heating rate was slower than the resistance heating of the NPS flow cell. This turned out to be a real challenge in the DPPC and DSPC analysis where, despite thorough degassing, bubbles began to form when the system reached over 50 °C while the heating rate got even slower. In an attempt to overcome the mentioned issues, an NPS flow cell was used as an alternative means of heating the transported solution and it was connected in series with the polytetrafluorethylene QCM flow cell. Due to expected cooling of the transported solution in the non-tempered QCM cell, the temperature range for DPPC was extended up to 60 °C. However, no sudden changes of frequency were detected during the adsorption of DPPC liposomes even with prolonged rinsing with liposomal dispersion at 60 °C (data not shown).

This data actually suggests that QCM is a rather inconvenient methodology for the analysis of lipids with higher T_m_ values. On the other hand, Peltier cooling/heating made the temperature gradient of the QCM analysis of DMPC more repeatable than the NPS manual cooling. However, this did not help to obtain a well-defined change in the signal, suggesting phase transition of adsorbed liposomes. The above results indicate that while QCM is generally considered as a complementary technique to NPS, in this particular case, it is not able to analyze T_m_ values during online analysis.

**References**

1.     Ohlsson, G., Tigerstrom, A., Höök, F. & Kasemo, B. Phase transitions in adsorbed lipid vesicles measured using a quartz crystal microbalance with dissipation monitoring. *Soft Matter* **7**, 10749-10755 (2011).

2.     Seantier, B., Breffa, C., Félix, O. & Decher, G. In situ investigations of the formation of mixed supported lipid bilayers close to the phase transition temperature. *Nano Lett.* **4**, 5-10 (2004).

3.     Wargenau, A. & Tufenkji, N. Direct detection of the gel-fluid phase transition of a single supported phospholipid bilayer using quartz crystal microbalance with dissipation monitoring. *Anal. Chem.* **86**, 8017-8020 (2014).
